# Repeat cross-sectional data on the progression of the metabolic syndrome in Ossabaw miniature swine

**DOI:** 10.1016/j.dib.2016.04.023

**Published:** 2016-04-13

**Authors:** Mikaela L. McKenney-Drake, Stacey D. Rodenbeck, Meredith K. Owen, Kyle A. Schultz, Mouhamad Alloosh, Johnathan D. Tune, Michael Sturek

**Affiliations:** a Department of Cellular & Integrative Physiology, Indiana University School of Medicine, 635 Barnhill Dr., Indianapolis, IN 46202, United States; b College of Pharmacy & Health Sciences, Butler University, 4600 Sunset Avenue, Indianapolis, IN 46208, United States; c Covance, Inc. 671 South Meridian Road, Greenfield, IN 46140, United States

## Abstract

Ossabaw miniature swine were fed an excess calorie, atherogenic diet for 6, 9, or 12 months. Increased body weight, hypertension, and increased plasma cholesterol and triglycerides are described in Table 1. For more detailed interpretations and conclusions about the data, see our associated research study, “Biphasic alterations in coronary smooth muscle Ca^2+^ regulation during coronary artery disease progression in metabolic syndrome” McKenney-Drake, et al. (2016) [1].

## Specification Table

**Table:** 

Subject area	*Physiology*
More specific subject area	*Metabolic syndrome development*
Type of data	*Table*
How data was acquired	*Plasma biochemical analysis*
Data format	*Analyzed*
Experimental factors	*Metabolic syndrome was induced by atherogenic diet feeding for 6, 9, and 12 months.*
Experimental features	*Repeat cross sectional study of metabolic syndrome induction at different time points of atherogenic diet feeding.*
Data source location	*Indianapolis, IN, United States of America.*
Data accessibility	*With this article*

## Value of the data

• These data could assist researchers in study design for induction of metabolic syndrome.
• Provide previously unreported time-dependent aspects of metabolic syndrome.
• May provide insight toward development of therapies at different time points of metabolic syndrome progression.

## 1 Data

Here, we conducted a repeat cross-sectional analysis of metabolic syndrome development in Ossabaw swine during atherogenic diet feeding for 6, 9, and 12 months, as described in the associated research study [1]. Ossabaw swine on atherogenic diet had increased body weight, hypertension, and dyslipidemia, compared to lean controls (Table 1).

## 2 Experimental design, materials and methods

### 2.1 Animal care

All experimental procedures involving animals were approved by the Institutional Animal Care and Use Committee at Indiana University School of Medicine with the recommendations outlined by the National Research Council and the American Veterinary Medical Association Panel on Euthanasia [2,3]. Six month old Ossabaw miniature swine were fed 1 kg of an excess-calorie atherogenic diet (KT-324, Purina Test Diet, Richmond, IN; 16% kcal from protein, 41% kcal from complex carbohydrates, 19% kcal from fructose, and 43% kcal from fat). The feed was supplemented with cholesterol (2.0%), hydrogenated coconut oil (4.70%), hydrogenated soybean oil (8.40%), cholate (0.70%), and high fructose corn syrup (5.0%) by weight [6], [7], [8], [5], [4] daily for 6 (*n*=6), 9 (*n*=7), or 12 (*n*=9) months. Lean control swine (*n*=9) were fed 725 g of a standard diet (5L80, Purina Test Diet, Richmond, IN; 18% kcal from protein, 71% kcal from complex carbohydrates, and 11% kcal from fat). Swine were housed individually with free access to drinking water and on a 12 h light/dark cycle.

### 2.2 Metabolic phenotyping

Final body weights and blood were obtained at time of sacrifice. Plasma was obtained from heparinized whole blood by centrifugation at 2000 rpm for 20 min. Lipid and glucose biochemistry was performed by ANTECH Diagnostics (Fishers, IN).

### 2.3 Statistical analysis

Statistical analysis was performed using GraphPad Prism 5.0 (San Diego, CA). One-way analysis of variance (ANOVA) with Bonferroni post hoc analysis was performed. Data are represented as mean±SEM. *p*<0.05 was considered significant.

## Figures and Tables

**Table 1:** Metabolic characteristics of Ossabaw miniature swine groups.

	**Lean**	**MetS (6 months)**	**MetS (9 months)**	**MetS (12 months)**	**Significance**
**Body weight (kg)**	62±5	89±2	87±7	116±2	12>9, 6>lean
**Fasting blood glucose (mg/dL)**	84±6	75±2	82±7	81±2	NS
**Systolic blood pressure (mmHg)**	131±7	150±9	143±4	170±7	12, 9, 6>lean
**Diastolic blood pressure (mmHg)**	63±2	77±5	85±4	89±5	12, 9>6, lean
**Total cholesterol (mg/dL)**	57±5	383±39	546±66	247±17	9>12, 6>lean
**Triglycerides (mg/dL)**	25±4	34±4	98±34	43±6	9>12, 6, lean

NS=not significant.
