# Supplementary material for: Repeat cross-sectional data on the progression of the metabolic syndrome in Ossabaw miniature swine
Source: Data Brief. 2016 Apr 13;7:1393–5. doi: 10.1016/j.dib.2016.04.023 (PMC4845153; doi:10.1016/j.dib.2016.04.023)
Supplement: Supplementary file 1 — Supplementary material [file mmc1.doc]

**Conflict of Interest**

We have no conflicts of interest regarding financial support of the work or involvement in for-profit companies.


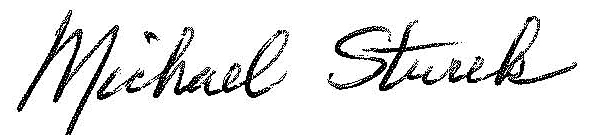


Michael Sturek, Ph.D.

Professor and Chair of Cellular & Integrative Physiology

Professor of Biomedical Engineering, Purdue University

[msturek@iu.edu](mailto:msturek@iu.edu)
